# PLOD2 Is a Potent Prognostic Marker and Associates with Immune Infiltration in Cervical Cancer

**DOI:** 10.1155/2021/5512340

**Published:** 2021-06-28

**Authors:** Guang Li, Xuefeng Wang, Guobing Liu

**Affiliations:** ^1^Department of Obstetrics and Gynecology, Third Affiliated Hospital of Southern Medical University, Guangzhou, 510515 Guangdong Province, China; ^2^Department of Obstetrics and Gynecology, NanFang Hospital of Southern Medical University, Guangzhou, 510515 Guangdong Province, China

## Abstract

**Background:**

PLOD2 is overexpressed in diverse tumors and plays a vital role in tumorigenesis. However, the prognostic value of PLOD2 in cervical cancer (CESC) remains unclear.

**Methods:**

PLOD2 expression and CESC patients' survival data were collected from the Oncomine, GEPIA, UALCAN, and Kaplan-Meier Plotter databases; immunohistochemistry (IHC) was used to validate the expression of PLOD2 in CESC; Gene Set Enrichment Analysis was performed using the STRING and DAVID databases; and the correlations between PLOD2 and cancer immune infiltrates were investigated using the TIMER and TISIDB databases.

**Results:**

We found that the expression level of PLOD2 was increased in various cancers, and meta-analysis in the Oncomine database revealed that PLOD2 was significantly upregulated in CESC compared to that in normal tissues (*P* < 0.001). In addition, the high expression of PLOD2 was closely related to poor overall survival (OS) and disease-free survival (DFS) in patients with CESC (OS HR = 1.73, *P* = 0.029; DFS HR = 2.60, *P* = 0.018). Functional annotations indicated that differentially expressed PLOD2 were primarily related to protein digestion and absorption pathways and to the collagen fibril organization process. Immune infiltration analysis showed that PLOD2 was highly correlated with B cells, CD4+ T cells, T helper type 2 (Th2) cells, and eosinophils in CESC.

**Conclusion:**

PLOD2 is positively associated with poor prognosis and might be considered a novel diagnostic and prognostic marker for CESC patients.

## 1. Introduction

Cervical cancer is the fourth most common malignancy in women worldwide. There are over 520 thousand new CESC cases and over 260 thousand deaths per year, with over 85% of these occurring in developing countries [1]. The main cause of CESC is the infection of HPV, but the infection itself is not enough to induce malignant transformation. The activation of the protooncogene and the inactivation of the tumor suppressor gene play an important role in the occurrence of CESC. Great progress has been made in targeted therapy in treating CESC patients, and patients with advanced or recurrent CESC treated with these therapies could survive for a longer period than before. In 2015, the NCCN guidelines recommended a first-line chemotherapy regimen for the VEGF inhibitor bevacizumab for recurrent CESC because it significantly prolonged a patient's survival time (17.0 months vs. 13.3 months *P* = 0.004) [2]. Besides, remarkable progress has been made in other new genes, like epidermal growth factor receptor blocker (EGFR) and poly-ADP-ribose polymerase (PARP) inhibitors. However, this is far from sufficient, and more therapeutic targets and prognostic biomarkers must be identified.

Procollagen lysine,2-ketoglutarate 5-dioxygenase (PLOD), also known as lysyl hydroxylase, is a protein encoded by the PLOD gene, playing an important role in fibrotic processes and tissue remodeling by activating hydroxylation of collagen fiber molecules [3]. There are three isoforms of PLOD, namely, PLOD1, PLOD2, and PLOD3, among which PLOD2 is essential for the biogenesis of normal mature collagen, tissue remodeling, and the stability of collagen crosslinks [3]. The overexpression of PLOD2 can promote the crosslinking of collagen, increase the hardness of the extracellular matrix, and promote the proliferation and metastasis of tumor cells [4]. Upregulation of PLOD2 has been observed in various human malignancies, including breast cancer [5], biliary cancer [6], colorectal cancer [7], glioma [8], and liver cancer [9], as well as in CESC [10, 11]. Although these studies have provided some useful information, they were limited by many factors, such as the small quantities of tumor samples and the lack of survival analysis data and related pathway research. In this study, we are going to evaluate the expression, prognosis, pathway, and immune infiltration of PLOD2 in CESC by immunohistochemistry and bioinformatics.

In this study, we analyzed the expression of PLOD2 and its correlation with the prognosis in CESC patients. Subsequently, functions and signal pathway enrichments of PLOD2 and related genes were analyzed. Finally, we studied the correlation between PLOD2 and tumor-infiltrating immune cells in the tumor microenvironment. The results in this study revealed the critical role of PLOD2 in CESC, and it may serve as a potent prognostic marker and be associated with immune infiltration in cervical cancer.

## 2. Materials and Methods

### 2.1. Oncomine Database Analysis

Oncomine (http://www.oncomine.org/) is a large-scale tumor gene chip database that has strong analytical ability and can be used to calculate gene expression characteristics, clustering, and genomic modules [12]. Data were extracted from Oncomine to evaluate the expression of PLOD2 in CESC. In this study, a *P* value of 1*E*‐4, a fold change of 2, and a gene rank in the top 10% were set as the significance thresholds; Student's *t*-test was used to analyze the difference in the expression of PLOD2 in CESC.

### 2.2. GEPIA Database Analysis

The GEPIA database (http://gepia.cancer-pku.cn/index.html) is a public database used to study gene expression differences in cancer and normal tissues, including 9736 tumors and 8587 normal samples of RNA sequencing expression data from TCGA and GTEX projects [13]. We performed a differential mRNA expression analysis of tumor and normal tissues and correlative prognostic analysis of PLOD2 via the “Single Gene Analysis” module. The *P* value was 0.05, Student's *t*-test was used to generate a *P* value for expression analysis, and prognostic analysis was performed using a Kaplan-Meier curve.

### 2.3. IHC Staining and Evaluation

The study protocol was approved by the Medical Ethics Committee of NanFang Hospital, Southern Medical University (NFEC-2020-076); all patients had signed an informed consent before surgery; human tissues were collected from January 2018 to January 2019 at the NanFang Hospital, Southern Medical University; IHC was done in 19 normal cervical tissues and 15 CESC tissues; and a rabbit anti-PLOD2 antibody (bs-12731R, BIOSS, China) was used for IHC.

The expression of PLOD2 protein in tissues was measured by a semiquantitative method. In each sample, the percentage and intensity of tumor cells stained with PLOD2 in three random fields were evaluated by a 400 × lens. The IHC scoring was based on the percentage of immunoreactive tumor cells (negative = 0; <25% = 1; 25‐50% = 2; 51‐75% = 3; and >75% = 4) and intensity of immunoreactivity (negative = 0; light yellow = 1; yellow = 2; and dark yellow or brown = 3).

### 2.4. Kaplan-Meier Plotter Database

The prognostic value of PLOD2 mRNA expression in CESC was assessed by overall survival (OS) and relapse-free survival (RFS) using the Kaplan-Meier Plotter (http://www.kmplot.com) [14], an online database including gene expression data and clinical data. In order to assess the prognostic value of a specific gene, the patient samples were divided into two cohorts according to the expression of the gene (high vs. low expression), and logrank *P* values and HRs with 95% confidence intervals were determined on the webpage.

### 2.5. Bioinformatic Analysis and Functional Enrichment

STRING (https://string-db.org/) is aimed at collecting, scoring, and integrating all publicly available sources of protein-protein interaction (PPI) data, and it complements these with computational predictions of potential functions [15]. We conducted a PPI network analysis of PLOD2 and the neighboring 10 genes significantly associated with it by using STRING.

DAVID 6.8 (http://david.abcc.ncifcrf.gov/), a common functional annotation tool of bioinformatics resources was utilized to distinguish the biological function of submitted genes [16]. In our study, the Gene Ontology (GO) enrichment analysis and Kyoto Encyclopedia of Genes and Genomes (KEGG) pathway enrichment analysis of PLOD2 and closely related neighbor genes were isolated from DAVID 6.8 and visualized by Omicshare (http://www.omicshare.com/). Biological processes (BP), cellular components (CC), and molecular function (MF) were included in GO enrichment analysis.

### 2.6. Immune Infiltration Analysis

The TIMER (https://cistrome.shinyapps.io/timer/) database contains 32 cancers from The Cancer Genome Atlas (TCGA) with 10897 samples [17], and it was used to perform comprehensive correlation analysis between tumor-infiltrating immune cell signatures and PLOD2 genes. The TISIDB database (http://cis.hku.hk/TISIDB/index.php) integrates 988 reported immune-related antitumor genes, high-throughput screening techniques, molecular profiling, and paracancerous multiomics data, as well as various resources for immunological data retrieved from seven public databases [18]. In our study, the TISIDB database was used to investigate correlations between PLOD2 expression and lymphocytes.

### 2.7. Western Blot

The total cellular protein was extracted by RIPA Lysis Buffer, and the protein concentrations of the cell lysates were measured using a Pierce™ BCA Protein Assay Kit (Thermo Fisher Scientific) and equalized before loading. Equal amounts of protein extracts from Hela cells and HUCEC cells were separated by SDS-PAGE and transferred onto polyvinylidene fluoride membranes (Sigma-Aldrich, MO, USA). Immunoblot analyses were carried out using the appropriate antibodies (Solarbio, Beijing, China).

## 3. Result

### 3.1. PLOD2 DNA Expressed in CESC

**Figure 1:**
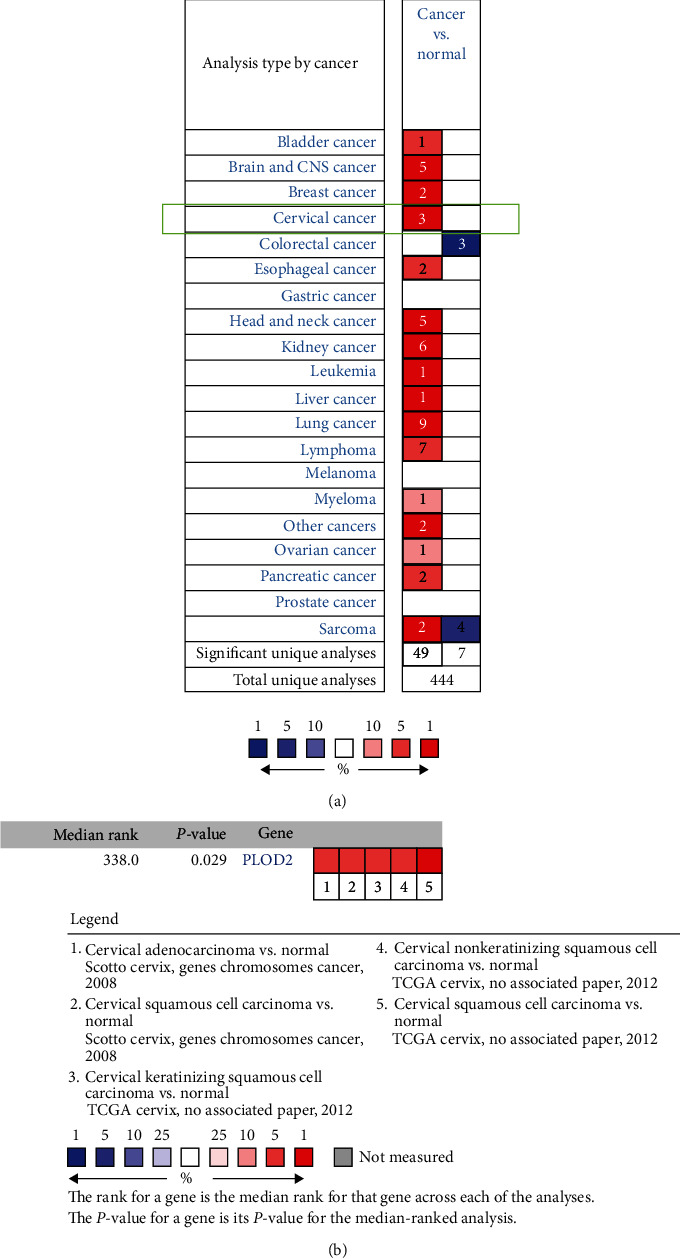
(a) The expression pattern of PLOD2 in different cancer types; PLOD2 expression is significantly higher in CESC tissues than in normal tissues. (b) Meta-analysis of PLOD2 DNA expression in 5 analyses.

We initially used the Oncomine database to analyze the differential DNA expression level of PLOD2 between CESC and normal tissues. As depicted in Figure 1(a), the database contained a total of 444 unique analyses of PLOD2, with a total of 3 studies demonstrating a significantly increased expression level of PLOD2 in CESC. For validation, we performed meta-analysis of PLOD2 DNA expression in 5 analyses with a threshold using a *P* value ≤ 0.05, fold change ≥ 2, and top 10% gene rank in the Oncomine database. As shown in Figure 1(b), compared with that in normal tissues, PLOD2 was upregulated in cervical cancer (*P* < 0.05, Figure 1(b)).

### 3.2. Expression of PLOD2 in CESC

**Figure 2:**
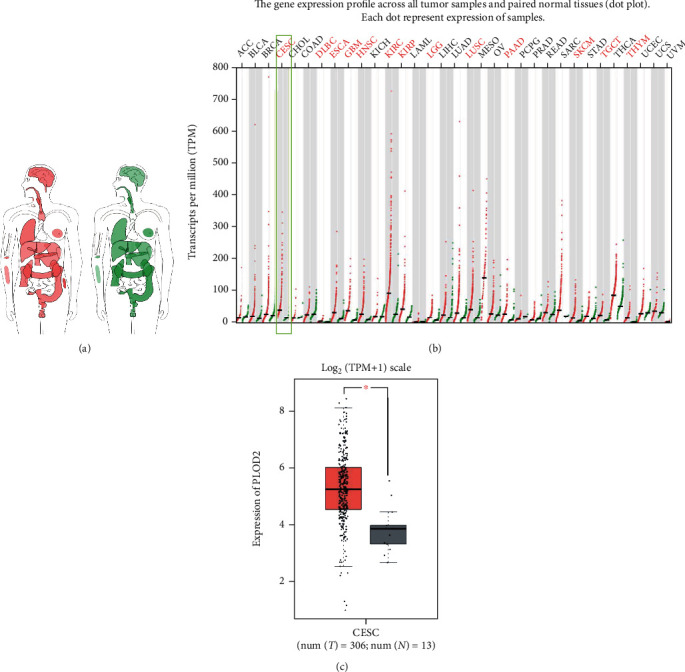
Expression of PLOD2 in cancer and normal tissues from GEPIA. (a) PLOD2 median expression of tumor (red) and normal (green) samples in a bodymap. (b) PLOD2 expression profile across all tumor (red) and paired normal (green) tissues. (c) The expression of PLOD2 mRNA in CESC tissues (red box) and paired normal tissues (black box) from GEPIA. ^∗^*P* < 0.05; ^∗∗^*P* < 0.001; ^∗∗∗^*P* < 0.0001.

**Figure 3:**
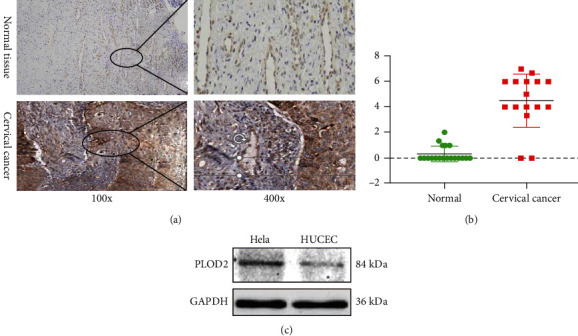
(a) IHC staining of PLOD2 protein in normal cervical tissue and CESC. (b) IHC score analysis of PLOD2 between CESC and normal tissues (^∗^*P* < 0.05; ^∗∗^*P* < 0.001; ^∗∗∗^*P* < 0.0001). (c) Expression of PLOD2 between Hela cells and normal cervical cells.

The GEPIA dataset was used to compare the mRNA expression of PLOD2 in CESC tissues and normal cervical tissues. According to our findings, the PLOD2 mRNA expression level was upregulated in CESC tissues relative to that in normal tissues (Figures 2(a) – 2(c)*P* < 0.05). Then, we confirmed by Western blot that the expression of PLOD2 in the cervical cancer cell line was higher than that in normal cervical cells (Figure 3(c)).

### 3.3. Pathological Significance of PLOD2 Expression in CESC

To analyze the clinicopathologic roles of PLOD2 in CESC, 34 samples were examined via IHC, including 19 normal cervical tissue samples and 15 CESC tissue samples. PLOD2 was detected in the cytoplasm with higher expression in tumors compared with normal tissues. The immunohistochemical score of CESC tissues was significantly higher than normal ones (0.32 ± 0.58 vs. 4.50 ± 2.13, *P* < 0.001; Figures 3(a) and 3(b)).

### 3.4. The Prognostic Value of PLOD2 in Patients with CESC

The CESC dataset from TCGA was used to determine the independent prognostic potential of PLOD2 expression for OS by univariate and multivariate Cox regression analyses (Table 1). These results indicated that high levels of PLOD2 expression may lead to poor prognosis in patients with CESC.

Furthermore, to evaluate the value of differentially expressed PLOD2 in the progression of CESC, we assessed the correlation between differentially expressed PLOD2 and clinical outcome by the Kaplan-Meier Plotter. Patients with higher PLOD2 expression have significantly worse overall survival (OS) compared to patients with lower PLOD2 expression (Figure 3(a); HR = 1.73, 95%CI = 1.05 to 2.84, *P* = 0.029); relapse-free survival (RFS) was also lower in these patients, but the difference was not statistically significant (Figure 3(b); HR = 2.08, 95%CI = 0.94 to 4.95, *P* = 0.063).

**Figure 4:**
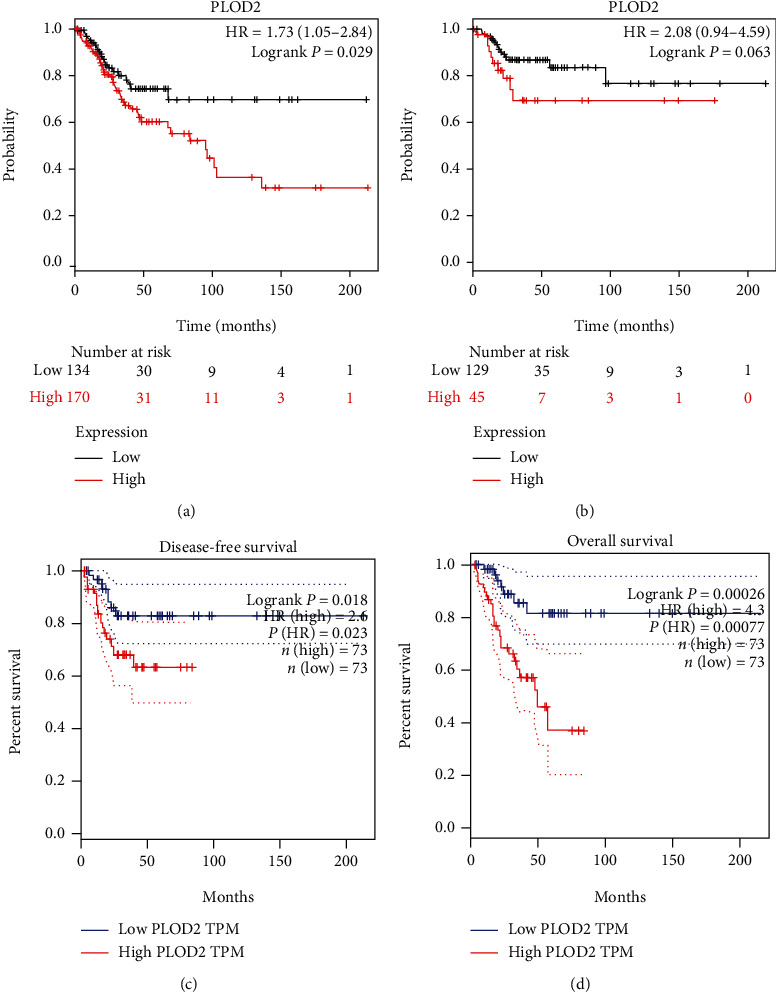
(a, b) High PLOD2 expression was correlated with poor OS in CESC and was not associated with RFS (Kaplan-Meier Plotter). (c, d) High PLOD2 expression was correlated with poor OS and RFS in CESC (GEPIA).

To further confirm the above results, we used RNA sequencing data from TCGA databases to analyze the prognostic potential of PLOD2 in CESC via CEPIA. High PLOD2 expression was associated with poor prognosis of OS and DFS in CESC (OS HR = 4.3, *P* < 0.001; DFS HR = 2.6, *P* = 0.023) (Figures 4(c) and 4(d)). These results confirmed the prognostic value of PLOD2 in CESC.

### 3.5. Functional Annotations and Predicted Signaling Pathways

**Figure 5:**
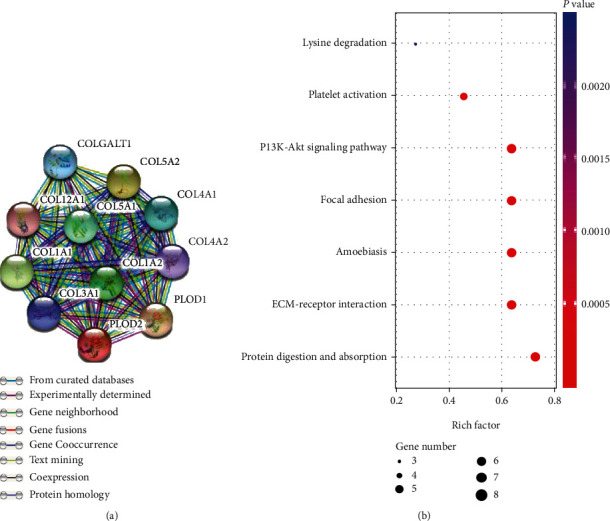
(a) Protein-protein interaction network construction using PLOD2 and significantly correlated genes. (b) Enrichment results of KEGG signal pathways.

**Figure 6:**
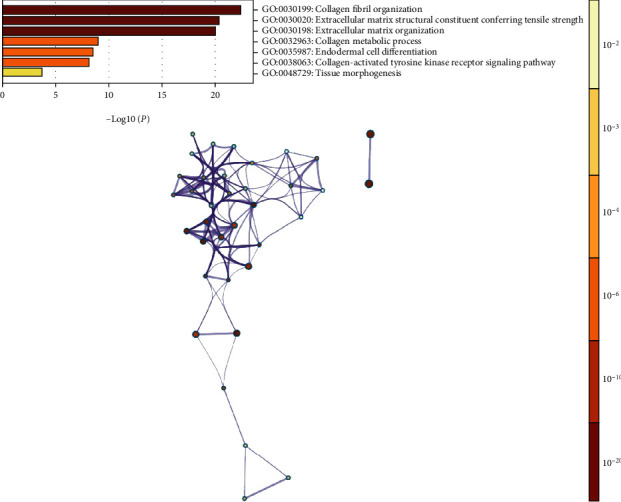
Enrichment results of GO term.

A PPI network was constructed using 10 genes identified from the STRING database significantly associated with PLOD2, and the results showed that COL5A2, COL4A1, COL3A1, PLOD1, COL12A1, COL5A1, COL1A2, COLGALT1, COL1A1, and COL4A2 were significantly related to PLOD2 (Figure 5(a)). Next, GO and KEGG analyses using DAVID 6.8 were exploited to discover the functional enrichment of PLOD2 and associated genes. As illustrated in Figure 5(b), GO enrichment analyses of 11 involved genes were visualized in a bubble chart, and the result shows that the processes below were subjected to the influence of the PLOD2 gene alteration (GO:0030199 collagen fibril organization; GO:0030020 extracellular matrix structural constituent conferring tensile strength; and GO:0030198 extracellular matrix organization) (Figure 6). KEGG pathway analyses were also performed, and according to our results, attention should be paid to some pathways including protein digestion and absorption, ECM-receptor interaction, amoebiasis, focal adhesion, PI3K-Akt signaling pathway, and platelet activation (Figure 5(b)).

### 3.6. Correlation between PLOD2 and Immune Infiltration

**Figure 7:**
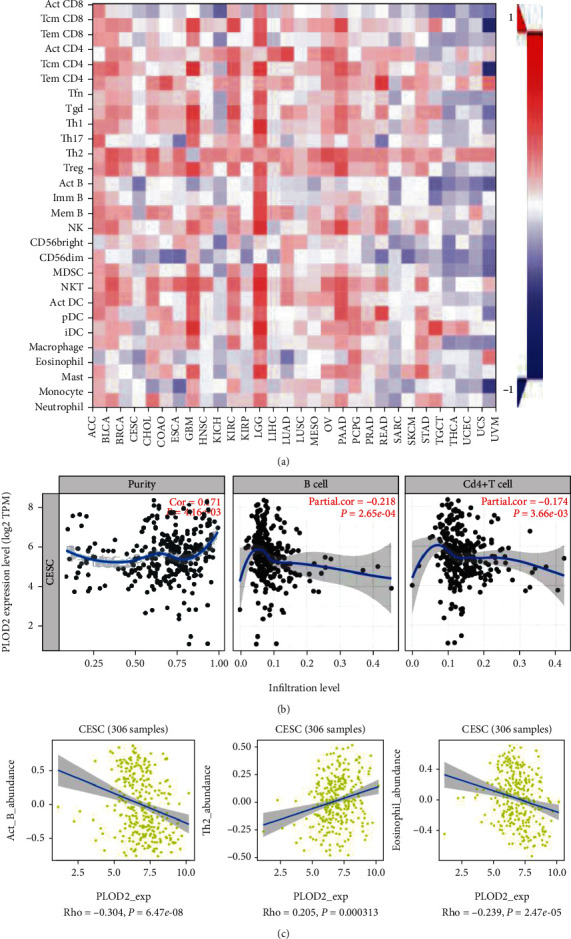
(a) Spearman's correlation of PLOD2 with lymphocytes and immunomodulators (TISIDB). (b) Relationship between the abundance of B cells and CD4+ T cells and PLOD2 expression. (c) Relationship between the abundance of ACT-B cells, Th2 cells, and neutrophils and PLOD2 expression.

Previous studies have shown that the occurrence and development of CESC are closely related to the tumor microenvironment. Cells in the microenvironment, such as adipocytes and fibroblasts, play an important role in tumor progression [19]; thus, we performed correlation analysis between PLOD2 and immune infiltration level in CESC. The result indicated that the expression of PLOD2 is positively correlated with the infiltration of the Th2 cell but negatively correlated with the infiltration of B cells, CD4+ T cells, and eosinophils (Figures 7(b) and 7(c)).

## 4. Discussion

PLOD gene family includes the PLOD1, PLOD2, and PLOD3 genes, which encode the LH1, LH2, and LH3 proteins, respectively. The main function of these proteins is promote the maturation and secretion of collagen by catalyzing the hydroxylation of lysine residues after the translation of procollagen molecules. PLOD2 was dysregulated in various malignant tumors and always associated with poor prognosis, but the role of PLOD2 in CESC has rarely been demonstrated.

Hypoxia-inducible factor 1 (HIF-1) activates transcription of the PLOD2 gene [20]; a recent microarray technique research conducted in 28 invasive CESCs and 5 normal cervix samples has demonstrated that PLOD2 was overexpressed in tumors relative to normal tissues [11]. We found the same results in 306 CESCs and 13 normal cervical samples by bioinformatics analysis, and 34 samples examined using IHC staining was consistent with the results above. Therefore, these indicated that the expression of PLOD2 may predict the prognosis of CESC.

We further investigated the prognostic value of PLOD2 in CESC using the Kaplan-Meier Plotter and GEPIA. The prognosis of PLOD2 has been reported in several human cancers before, but it has never been described in CESC. Noda et al. found that PLOD2 was highly expressed in liver cancer (*P* < 0.05), and DFS of HC patients in the high-expression group was significantly lower than that in the low-expression group (*P* = 0.002) [9]. Wei et al. found that in breast cancer, the high expression of PLOD2 could reshape collagen arrangement and promote the metastasis of breast cancer [21]. Moreover, the upregulation of PLOD2 could alter collagen crosslinking of tumor stroma and form aligned and stiff collagen fibers; these networks act as “highways” for tumor cells by supporting their scaffold and facilitating their migration towards blood vessels resulting ultimately to dissemination to distant sites [10]. In addition, the high expression of PLOD2 can also cause chemotherapy resistance of gemcitabine [22], while downregulating the expression of PLOD2 in BTC-GR cells can significantly improve the sensitivity to gemcitabine. In our study, the high expression of PLOD2 in CESC was significantly associated with worse OS and DFS in CESC patients. These findings collectively elucidated that the expression of PLOD2 might be a potential biomarker for prognosis of CESC.

A previous study has demonstrated that the PI3K/AKT-FOXP1 pathway plays a role in cervical cancer progression [23]. In our study, the KEGG pathway and GO biological process of PLOD2 and interactive genes revealed that PLOD2 is deeply involved in the protein digestion/absorption pathway and collagen fibril organization process. The two pathways mentioned above are closely related to the extracellular matrix (ECM), which is essential for tumor invasion and metastasis through biological barriers. This means that PLOD2 expression may promote the migratory, invasive, and adhesive capacities of cervical cancer cells by affecting ECM; this could be further researched through in vitro and in vivo experimental studies.

Increasing evidence suggests that immune cell infiltration could affect tumor progression and recurrence and act as a significant determinant of both response to immunotherapy and clinical outcome. In this study, we found a significant correlation between the expression of PLOD2 and the infiltration of B cells, CD4+ T cells, Th2 cells, and neutrophils, indicating that PLOD2 is not only a prognostic indicator but also a reflection of immune status.

There were some limitations to the current study. Firstly, the functions and involved pathways of PLOD2 were only evaluated by bioinformatics methods, and they need to be further validated by in vitro and in vivo experimental studies in future research. Secondly, the exact mechanisms of immune infiltration of PLOD2 remain to be fully elucidated; it will be another research point for us.

## 5. Conclusion

The current research systematically examined the expression of PLOD2 genes and its prognostic significance in CESC, which sheds more light on the complexity and heterogeneity of CESC biological properties. In summary, upregulation of PLOD2 in CESC probably exerts a crucial part during CESC oncogenesis. Besides, PLOD2 may serve as a potential prognostic factor in CESC patients. Collectively, these data suggest that PLOD2 is worthy of further study in CESC, and it may be a potential biomarker, which can be used to improve the survival rate and prognosis accuracy of CESC.

## Figures and Tables

**Table 1:** Univariate and multivariate Cox regression analyses of PLOD2 expression for overall survival (OS) in patients with cervical cancer from The Cancer Genome Atlas (TCGA) dataset.

Characteristics	Total (*N*)	Univariate analysis		Multivariate analysis	
		Hazard ratio (95% CI)	*P* value	Hazard ratio (95% CI)	*P* value
Clinical stage (stage III and stage IV vs. stage I and stage II)	302	2.329 (1.432-3.788)	**<0.001**	1.543 (0.762-3.126)	0.228
BMI (>25 vs. ≤25)	263	0.599 (0.348-1.033)	0.065	0.841 (0.433-1.631)	0.608
Birth control pill history (yes vs. no)	160	0.674 (0.325-1.398)	0.289		
Age (>50 vs. ≤50)	309	1.281 (0.805-2.037)	0.296		
Primary therapy outcome (CR vs. PD and SD and PR)	221	0.072 (0.039-0.133)	**<0.001**	0.069 (0.033-0.144)	**<0.001**

## Data Availability

The datasets of this study have mainly been collected, obtained, and analyzed from corresponding online databases, and other data generated or analyzed are available from the corresponding authors upon reasonable request.
