# Corrigendum to “PLOD2 Is a Potent Prognostic Marker and Associates with Immune Infiltration in Cervical Cancer”

**DOI:** 10.1155/2021/9762405

**Published:** 2021-10-23

**Authors:** Guang Li, Guobing Liu, Xuefeng Wang

**Affiliations:** ^1^Department of Obstetrics and Gynecology, Third Affiliated Hospital of Southern Medical University, Guangzhou, 510515 Guangdong Province, China; ^2^Department of Obstetrics and Gynecology, NanFang Hospital of Southern Medical University, Guangzhou, 510515 Guangdong Province, China

In the article titled “PLOD2 Is a Potent Prognostic Marker and Associates with Immune Infiltration in Cervical Cancer” [1], the authors wish to provide the following acknowledgement that was omitted in error:

This work was financially supported by the Natural Science Foundation of Guangdong Province (2021A1515011050) and Science and Technology Projects in Guangzhou (202102080100).

Additionally, the order of authors was listed incorrectly, and the correct order is as shown above.
